# Tunable Photoinduced Charge Transfer at the Interface between Benzoselenadiazole-Based MOF Linkers and Thermally Activated Delayed Fluorescence Chromophore

**DOI:** 10.1021/acs.jpcb.2c08844

**Published:** 2023-02-21

**Authors:** Shorooq A. Alomar, Luis Gutiérrez-Arzaluz, Issatay Nadinov, Ru He, Xiaodan Wang, Jian-Xin Wang, Jiangtao Jia, Osama Shekhah, Mohamed Eddaoudi, Husam N. Alshareef, Kirk S. Schanze, Omar F. Mohammed

**Affiliations:** † Advanced Membranes and Porous Materials Center and KAUST Catalysis Center, Division of Physical Science and Engineering, King Abdullah University of Science and Technology (KAUST), Thuwal 23955-6900, Kingdom of Saudi Arabia; ‡ Functional Materials Design, Discovery and Development Research Group (FMD), Advanced Membranes and Porous Materials Center (AMPMC), Division of Physical Sciences and Engineering (PSE), King Abdullah University of Science and Technology (KAUST), Thuwal 23955-6900, Kingdom of Saudi Arabia; § Materials Science and Engineering, Division of Physical Sciences and Engineering (PSE), King Abdullah University of Science and Technology (KAUST), Thuwal 23955-6900, Kingdom of Saudi Arabia; ∥ Department of Chemistry, University of Texas at San Antonio, San Antonio, Texas 78249, United States

## Abstract

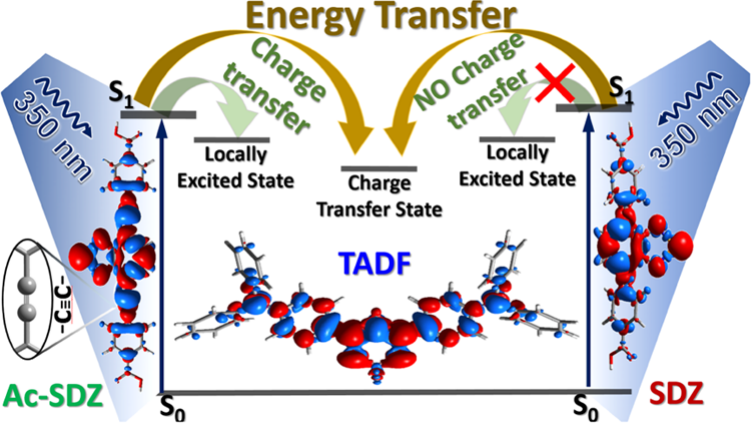

Structural modifications to molecular systems that lead to the control of photon emission processes at the interfaces between photoactive materials play a key role in the development of fluorescence sensors, X-ray imaging scintillators, and organic light-emitting diodes (OLEDs). In this work, two donor–acceptor systems were used to explore and reveal the effects of slight changes in chemical structure on interfacial excited-state transfer processes. A thermally activated delayed fluorescence (TADF) molecule was chosen as the molecular acceptor. Meanwhile, two benzoselenadiazole-core MOF linker precursors, Ac-SDZ and SDZ, with the presence and absence of a C≡C bridge, respectively, were carefully chosen as energy and/or electron-donor moieties. We found that the SDZ -TADF donor–acceptor system exhibited efficient energy transfer, as evidenced by steady-state and time-resolved laser spectroscopy. Furthermore, our results demonstrated that the Ac-SDZ–TADF system exhibited both interfacial energy and electron transfer processes. Femtosecond-mid-IR (fs-mid-IR) transient absorption measurements revealed that the electron transfer process takes place on the picosecond timescale. Time-dependent density functional theory (TD-DFT) calculations confirmed that photoinduced electron transfer occurred in this system and demonstrated that it takes place from C≡C in Ac-SDZ to the central unit of the TADF molecule. This work provides a straightforward way to modulate and tune excited-state energy/charge transfer processes at donor–acceptor interfaces.

## Introduction

The understanding of the relationship between the chemical structures of organic molecules and their optical properties is a very important aspect that has been explored for several years to produce new systems with enhanced photoluminescence (PL) or photochemical properties.^1,2^ However, the ability to fine-tune and control this kind of process in organic molecular systems is still difficult to achieve. One reason is the lack of understanding of which specific chemical structure features lead to the emissive channel.^3,4^ For that reason, it is crucial to understand the influence of structural modifications on excited-state dynamics at the atomic level to successfully achieve full control of the outcomes of photoinduced and deactivation processes of molecular systems.

One interesting family of chromophores that involves the control of different excited states is thermally activated delayed fluorescence (TADF) molecules, which have attracted substantial attention as luminescent materials due to their unique ability to harvest often detrimental triplet states as emissive singlet states through the reverse intersystem crossing process.^5−13^ This phenomenon prolongs the fluorescence lifetime of TADF materials from nanoseconds to microseconds,^14,15^ making them excellent candidates for several applications as luminescence sensors,^15−19^ organic light-emitting devices (OLEDs),^5,9,12,20^ and more recently, as X-ray imaging materials.^20−24^ However, due to the intricate interplay between the triplet and singlet excited states in TADF, it is challenging to control the optical properties of TADF materials by direct structural modifications, but they can serve as excellent energy and/or electron acceptor units.^2,5,8,11,18,19,25−27^ Other promising luminescent materials are heterobenzodiazole rings, which have also demonstrated excellent luminescent and photochemical behaviors.^28−34^ Unlike TADF materials, the structures of these organic linkers can be easily altered to achieve different reaction outcomes upon light excitation, and they can serve as energy/electron donors in a variety of chemical composites.^28,32,35^ Moreover, this kind of organic linker is designed to be an easy building block of metal–organic frameworks (MOFs)^36−39^ that can serve not only to improve the optical properties of MOFs but also to favor and increase the efficiency of energy transfer processes at the donor–acceptor interface due to the highly ordered structure of the frameworks. These properties have been exploited in sensing,^27,40−43^ catalysis,^44−47^ and light-harvesting applications, including X-ray imaging scintillation.^28,37,40^

**Figure 1:**
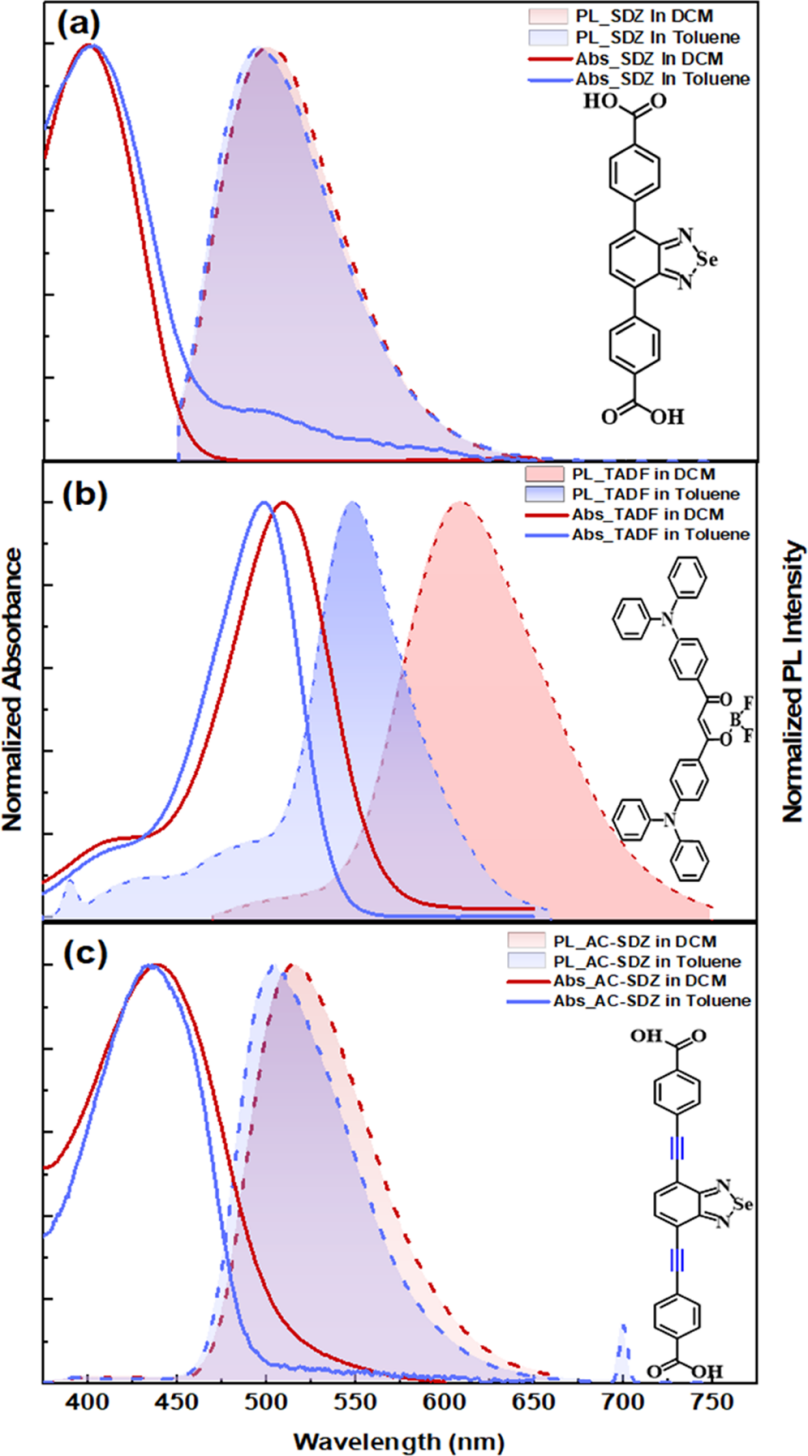
Normalized absorption and fluorescence spectra for (a) SDZ, (b) TADF, and (c) Ac-SDZ in different solvents (toluene/DCM), λ_exc_ = 350 nm. The peak at 380 nm is related to Raman scattering, and the peak at 700 nm is due to second-order diffraction of the excitation light at 350 nm.

In this study, we combined in a single composite the structural versatility of organic MOF linkers with a highly emissive TADF molecule to investigate and decipher the key structural elements that regulate energy and charge transfer processes at the interface of this system. We selected the TADF molecule as the energy/charge acceptor and two benzoselenadiazole linkers, with (Ac-SDZ) and without (SDZ) an acetylenic bridge (C≡C), as donors (see Figure 1). Using steady-state and time-resolved spectroscopic techniques, we confirmed the different photochemical behaviors of the two systems caused by C≡C. More specifically, the SDZ–TADF system underwent a distinct energy transfer process from SDZ to the TADF molecule, which took place on the order of hundreds of picoseconds, as evidenced by the PL quenching of SDZ and the PL enhancement of the TADF molecule. Moreover, the introduction of a C≡C bond between the aromatic moieties in the donor structure resulted in an additional charge transfer in the Ac-SDZ–TADF system, which was noticeable in the PL spectra and was confirmed by fs-mid-IR transient absorption measurements. More specifically, we found that the C≡C vibration in Ac-SDZ underwent a bathochromic shift from 2060 to 2053 cm^–1^, indicating the redistribution of electron density along this chemical bond and the weakening of its triple bond character. More importantly, for this system, we clearly observed a decay signal related to intermolecular charge transfer during the first picoseconds. Time-dependent density functional theory (TD-DFT) calculations agreed well with the photoinduced charge transfer phenomena in the Ac-SDZ–TADF system, clearly indicating that it takes place from the C≡C bond in Ac-SDZ to the central unit in the TADF molecule. These findings provide a better understanding of the minimal key structural elements that not only lead to more efficient and precise control of interfacial photochemical processes but can also help successfully tailor molecular systems to produce more efficient optical devices such as sensors, OLEDs, and X-ray imaging scintillators.

## Methods

### Synthesis

#### 4,4′-(Benzo[*c*][1,2,5]selenadiazole-4,7-diyl)dibenzoic Acid (SDZ)

This linker was purchased from SUNGYOUNG Chemical Limited Company and used without any further purification.

#### 4,4′-(Benzo[*c*][1,2,5]selenadiazole-4,7-diylbis(ethyne-2,1-diyl))dibenzoic Acid (Ac-SDZ)

Details concerning the synthesis and structural characterization of the compound are provided in the Supporting Information.

#### 4,4′-(2,2-Difluoro-2H-4-dioxaborinine-4,6-diyl)*bis*(N,N-Diphenylaniline) (TADF)

The synthesis and structural characterization of the compound are provided in the Supporting Information.

### Steady-State PL and Absorption Measurements

For the solutions, 0.94 mg of SDZ or 1.0 mg of Ac-SDZ was dissolved in 2 mL of dimethylformamide (DMF) and mixed with a dichloromethane (DCM) solution (5 μL in 2 mL of DCM). TADF was added to the solution gradually (2.5, 5, 7.5, 10, 12.5, 15, 17.5 μM) and measured after the solution reached equilibrium. The absorption spectra of the solutions were obtained in a 1 cm quartz cuvette in a Cary-5000 UV–vis spectrometer from Varian. PL measurements were performed with a Fluoromax-4 fluorimeter (Horiba).

### Time-Resolved Photoluminescence

Time-resolved photoluminescence (PL) data were obtained through the time-correlated single-photon counting (TCSPC) technique (Halcyone, Ultrafast Systems), and the corresponding excitation wavelength at 350 nm was selected using an optical parametric amplifier (Newport, Spectra-Physics) that was pumped with an Astrella femtosecond pulsed laser (800 nm, 150 fs, 1 kHz, Coherent). The photoluminescence signals for the donor and acceptor were collected, recollimated by a pair of parabolic mirrors, passed through a long-pass filter (420 nm, Newport), and finally focused on an optical fiber coupled to a monochromator and a photomultiplier tube (PMT) detector. The energy at each excitation wavelength was kept constant with the help of a pair of variable neutral density filters (Thorlabs). TCSPC histograms were fitted using the Lavenberg–Marquart algorithm implemented in Ultrafast System software. The overall time resolution for the system was estimated to be about 120 ps.

### Mid-IR Transient Absorption

Time-resolved mid-IR experiments were obtained by a Helios-IR spectrometer with wide broadband capability (Ultrafast Systems). Excitation pulses at 400 nm were generated using the second harmonic generation of a 150 fs Ti:sapphire regenerative amplifier operating at 1 kHz and 800 nm as fundamental light. The tunable wide-range mid-IR probe pulses were generated by difference-frequency mixing in a near-infrared optical parametric amplifier (Light Conversion). Mid-IR-transient absorption measurements were performed for Ac-SDZ and for the mixture of Ac-SDZ and TADF in a DMF solution, with the same concentration of 2 × 10^–3^ M for both compositions. The liquid samples were placed between two calcium fluoride cell windows with a spacer thickness of 300 μm. The cell was automatically rotated to ensure that a fresh portion of the sample was excited with each laser shot.

### DFT Calculations

Density functional theory (DFT) calculations and their time-dependent version (TD-DFT) were used for this work with the CAM-B3LYP functional^48^ and the 6-311G++(d,p) Popple’s basis set;^49^ the IEF-PCM approach^50^ was used to model the solvent as dichloromethane. The geometries of SDZ, Ac-SDZ, TADF, and negatively charged TADF were fully optimized in the ground state. Dimers formed by Ac-SDZ–TADF and SDZ–TADF were optimized from three initial geometries (see the Supporting Information), and the most stable ones were further explored. The minima were confirmed by frequency analysis. Later, the vertical excitations to the first singlet excited state were obtained to analyze the differences in electron density between the ground and excited states for all of the systems mentioned above. All calculations were performed using Gaussian 16 software,^51^ and density of states (TDOS) analysis was performed in the Multiwfn program.^52^

## Results and Discussion

To demonstrate the modulation of charge and energy transfer processes, two derivatives of benzoselenadiazole molecules (SDZ and Ac-SDZ in Figure 1a,c) were designed and synthesized as donors. These were selected due to their chemical structural similarities, as they share the same fused-ring core (see Figure 1). The addition of a C≡C bond has been proven to affect the excited-state dynamics of other similar molecules.^29,30^ To test the difference in their optical properties, we used both molecules SDZ and Ac-SDZ in two donor–acceptor systems with a TADF molecule (Figure 1b) to monitor changes in the luminescence properties during ground- and excited-state interactions.

### Steady-State and Time-Resolved Emission Measurements

As a first approximation, the optical behavior of the donors (SDZ, Ac-SDZ) and acceptor (TADF) molecules in different environments was investigated. The results in Figure 1 clearly illustrate the solvent polarity effects on the absorption and PL of the three molecules SDZ (Figure 1a), TADF (Figure 1b), and Ac-SDZ (Figure 1c) in two different solvents, toluene (ε = 2.38) and dichloromethane (DCM) (ε = 8.93). The absorption bands of SDZ in both solvents were closely spaced and positioned at ∼410 nm. Similarly, in Ac-SDZ, the polarity of the solvents slightly affected the position of the bands, which were centered at 430 nm. In contrast, the spectrum of TADF at 510 nm in a toluene solution shifted to 530 nm in DCM. This shift in TADF may be caused by various interactions, including solvation between the more polar solvent and the molecule.^6^ On the other hand, the emission peak of SDZ exhibited a slight change from 500 to 510 nm, which is common for molecules weakly interacting with a solvent. In the case of Ac-SDZ, as solvent polarity increased from toluene to DCM, the emission peaks shifted from 510 to 530 nm, indicating more stabilization of the emissive excited state in the more polar solvent. Finally, TADF showed a larger shift in emission from 560 to 620 nm. This change in spectra between different solvents could be attributed to greater stabilization of the charge transfer state due to charge delocalization in more polar solvents. In other words, such high stability can be attributed to the formation of a new state with strong charge transfer characteristics.^8^ It is important to mention that both selenadiazole-core molecules exhibit a tail in the absorption around 510 nm that can be attributed to aggregate formation, observed for this kind of molecules in nonpolar solvents. This feature in the absorption spectrum is dominated by aggregate formation rather than solvent polarity. The smaller SDZ molecule structure makes it more prone to coalesce in solution due to its stronger intermolecular interactions, like π–π stacking and H bondings.^29,33,34,42^

Once the steady-state optical properties of the isolated molecules were analyzed, and the TADF and Ac-SDZ molecules were found to be prone to forming charge transfer states, the next step was to study them as donor–acceptor (D–A) systems (SDZ–TADF and Ac-SDZ–TADF) in DCM solutions. UV–vis absorption and fluorescence spectra were measured under the same conditions for both systems to understand the effect of minor structural modification on the excited-state dynamics at the D–A interface.

**Figure 2:**
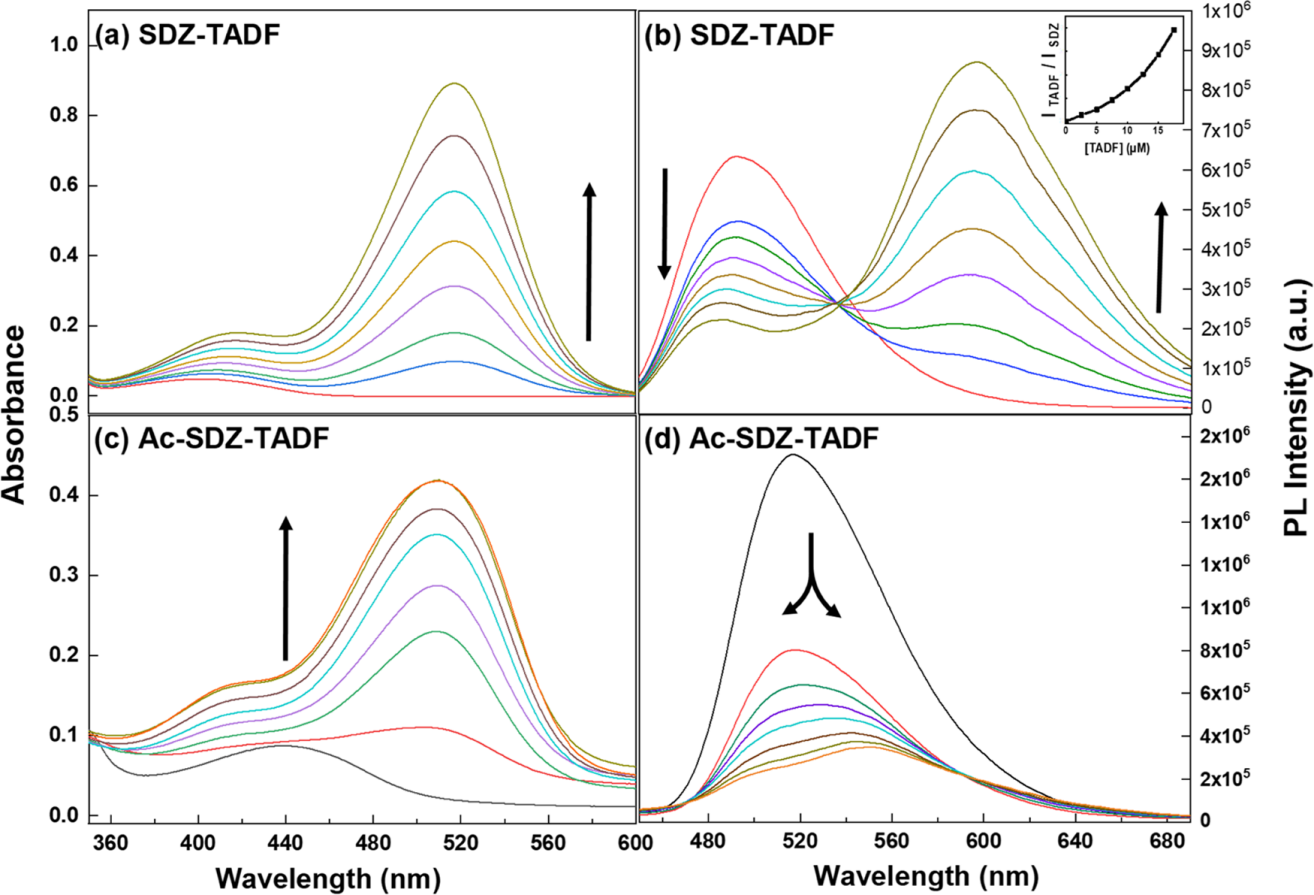
(a) Absorption and (b) emission spectra for SDZ in DCM and after the increase in the TADF concentration (inset is the corresponding emission intensity ratio between the emission maxima of TADF and SDZ). (c) Absorption and (d) emission spectra for molecular systems of Ac-SDZ in DCM and after the increases in the TADF concentration. λ_exc_ = 350 nm.

As shown in Figure 2a, in a dilute DCM solution, pure SDZ showed a broad absorption band over the range of 380–440 nm. After the TADF concentration was gradually increased (2.5–17.5 μM), a strong absorption band appeared at 520 nm. In contrast, the emission spectrum of pure SDZ was centered at 500 nm, as shown in Figure 2b. After the addition of the TADF acceptor, the SDZ emission showed strong quenching and a blue spectral shift to 490 nm, accompanied by an increase in the TADF PL intensity at ∼620 nm. It is important to mention that as the TADF concentration increased, the ratio between the PL intensities of TADF and SDZ also continuously increased (Figure 2b, inset), indicating that this enhancement in the TADF PL intensity is directly related to the decrease in SDZ PL intensity. These outcomes are in good agreement with typical energy transfer process behaviors observed from SDZ to TADF.^6,53^

For the Ac-SDZ–TADF system, the absorption spectrum of Ac-SDZ displayed a hypsochromic shift from 440 nm to 420 nm upon the addition of the TADF molecule (see Figure 2c), and the intensity of the absorption peak at 520 nm of the TADF molecule increased, as expected. Similarly, as shown in Figure 2d, a strong emission peak was observed corresponding to the pure donor at ∼520 nm. After the TADF molecule was added, the peak gradually split into two bands at 500 and 550 nm with quenching of their intensities. The emission band of the TADF molecule at 620 nm was also observed with a much lower intensity than in the SDZ–TADF system. These features, such as the presence of new emission bands and considerable PL quenching, could be related to intermolecular electron transfer from the donor molecule (Ac-SDZ) to the acceptor molecule (TADF), and locally excited-state formation.^6,18,29^ This observation provides another piece of evidence of the charge transfer process in this system. In other words, based on the absorption and emission spectroscopic measurements, we can assign the peak at 500 nm to the locally excited-state formation in Ac-SDZ caused by the intermolecular charge transfer phenomenon between Ac-SDZ and the TADF molecule.^15,18^ The formation of this locally excited-state band in Ac-SDZ (500 nm) is a consequence of the new charge density distribution after the charge transfer to the TADF molecule. Additionally, the second band around 550 nm is attributed to the original charge transfer state of Ac-SDZ molecule.^15−19^ From these steady-state measurements, we can summarize that the presence of a C≡C bond in the Ac-SDZ molecule can change its optical properties relative to the SDZ molecule in the presence of the acceptor, suggesting the important role of this bridge in controlling photochemical processes at the interface of the donor–acceptor system.

Time-resolved PL (TR-PL) spectra were measured for solutions with pure SDZ and TADF molecules and their mixture, detecting at the respective maxima (480, 620 nm; see Figure S7 and Table S1). The results showed a slight increase in the TADF molecule’s PL lifetime (from 1.23 ± 0.06 to 1.75 ± 0.02 ns) when the SDZ donor molecule was added, which could be attributed to energy transfer to the TADF molecule. On the other hand, the lifetime of SDZ decreased from 1.86 ± 0.04 ns to 224 ± 20 ps at the highest concentration of the TADF acceptor (17.5 μM). The dramatic decrease in the SDZ PL lifetime, together with the increase in the TADF molecule’s PL lifetime, is an indicator of energy transfer from the SDZ donor to the TADF acceptor.^53,54^ It should be noted that the slight increase in the TADF molecule’s PL lifetime relative to the decrease in the PL lifetime of SDZ can be explained by the presence of other deactivation channels in the TADF molecule that are also present after the transfer such as internal conversion, vibrational relaxation, and ISC.^5,7^ Finally, the TR-PL results for the SDZ–TADF system confirmed that energy transfer took place on the picoseconds timescale.

As shown in Figure 2d, the strong spectral overlap between the two emission bands generated during Ac-SDZ and TADF molecule emission makes it very difficult, if not impossible, to follow their isolated dynamics by TR-PL techniques. However, another time-resolved spectroscopic technique, mid-IR, can be very helpful in monitoring charge transfer dynamics by following the specific vibrational mode of the C≡C bond in the Ac-SDZ–TADF system in real time.

### Vibrational Spectroscopy

**Figure 3:**
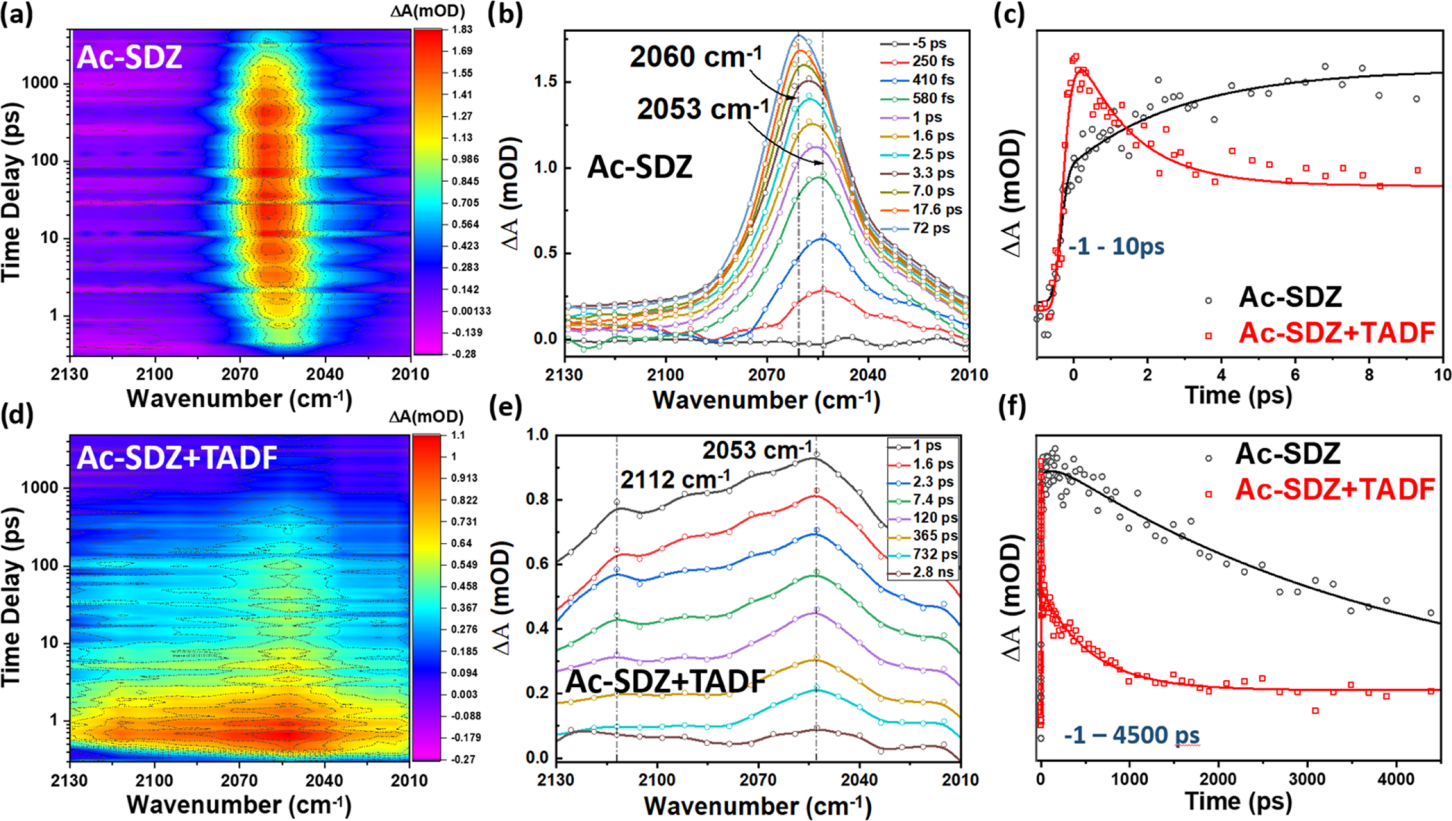
Time evolution of transient mid-infrared absorption as a function of wavenumber and intensity measured after excitation at 400 nm of (a) Ac-SDZ in DMF and (d) Ac-SDZ with TADF in DMF (the intensity scale was used as a contour plot). IR-transient absorption spectra at different time delays for (b) Ac-SDZ and (e) Ac-SDZ with TADF in DMF after 400 nm excitation. The kinetic traces at 2053 cm^–1^ for Ac-SDZ and for Ac-SDZ with TADF (c) at early times from −1 to 10 ps and (f) from −5 ps to 4.8 ns (solid lines represent their fit curves).

Considering the strong overlap between the donor and acceptor emission signals in the Ac-SDZ–TADF system, we used the exclusive advantages of mid-IR transient absorption spectroscopy to study the excited-state structural dynamics at the donor–acceptor interface by following a vibrational marker mode (C≡C) of the donor moiety. Unlike electronic spectroscopy, including fluorescence spectroscopy, the time-resolved-mid-IR technique is much more powerful in providing unique data about different local structural changes of molecules undergoing photoinduced processes in real time. Among the given molecules, it is easier to follow the excited-state dynamics of the C≡C stretching vibrations in Ac-SDZ and use those as a convenient probe and indicator for tracking excited-state reactions in the donor–acceptor system. This C≡C vibration mode is located in the spectral range of 2000–2250 cm^–1^ (see Figure 3b), which is isolated from other fingerprint vibrational modes, providing clear dynamics of the structural changes that occur upon light excitation.

According to our previous study, ground-state measurements for a molecule similar to Ac-SDZ but with a benzothiadiazole core show a peak for the C≡C band at 2205 cm^–1^ in DMF solution.^29^ Taking this into account, we used an Ac-SDZ solution in DMF to reach a strong C≡C vibrational signal from the fs-mid-IR technique. We confirmed that this change from DCM to DMF solvent does not affect the charge transfer process (see the Supporting Information, Figure S6). The peak position of the same band for Ac-SDZ was at ∼2205 cm^–1^ and was confirmed by the DFT calculations (see the Supporting Information, Figure S9). Figure 3a,b presents changes in the C≡C vibrational marker mode for Ac-SDZ in the excited state at different time delays (from 250 fs to 75 ps) at a concentration of 2 × 10^–3^ M in DMF. Early in the process (250 fs), the peak position was located at 2053 cm^–1^, which is a 152 cm^–1^ spectral downshift relative to the C≡C band in the ground state. It should be noted that the observed peak progressively shifted to a higher frequency, increasing from 2053 cm^–1^ to the maximum displacement at 2060 cm^–1^, along with a slow increase in the kinetic trace within a time constant of 80 ps (see Figure 3c, solid black line). Additionally, the kinetics of Ac-SDZ at 2055 cm^–1^ exhibited two vibrational mode relaxations. One of them presented a slow increase with a characteristic time constant of 4.6 ± 0.5 ps (31.8%) and slower decay of >4 ns (68.2%). The given time to achieve this increase can be associated with energy redistribution over the entire molecule after excitation, including through C≡C vibrations, and, as with other systems with similar chemical structures, it can be considered the time required for an aggregate formation and other associated photophysics,^29,45,55^ confirming that the origin of the tail observed in the steady-state absorption spectra is related to aggregation (Figure 1a,c). It should be noted that the C≡C bond plays an important role in excited-state aggregate formation, allowing the entire molecule to adopt a planar structure due to the low rotational barrier around the C≡C bond, which allows for easy stacking interactions between two molecules, as reported in another study involving a similar thiadiazole system.^29^

After exploring the Ac-SDZ excited-state dynamics, we studied the structural changes in Ac-SDZ (donor) after the addition of the TADF molecule (acceptor). IR-transient absorption measurements were performed for the mixture of solutions containing Ac-SDZ and the TADF molecule in DMF at a ratio of 1:2. Figure 3d,e displays the changes in the C≡C band at different time delays (from 250 fs to 4 ns) after excitation at 400 nm. The data in Figure 3d are completely different from those observed for the pure donor (Figure 3a) and reveal two significant distinctions. First, after excitation, we observed two major peaks. The first one is an intense peak at 2053 cm^–1^, and the second one is less intense at 2112 cm^–1^. These peaks exhibited a 58 cm^–1^ separation but with some spectral overlap. The big differences between the two peak positions can be associated with the charge transfer state formation. Second, these peaks displayed diverse dynamics, as extracted from the kinetic traces relative to the pure donor (see Figure 3c,f, solid red line). The kinetic trace measured at 2053 cm^–1^ exhibited three time constants of 1.73 ± 0.37 ps (55%), 500 ± 80 ps (32.9%), and >4 ns (12.1%). The first rapid decay at an early time (1.73 ps) could be interpreted as the electron transfer process from Ac-SDZ to the TADF molecule. The simultaneous formation of an additional IR feature at a higher wavenumber of 2112 cm^–1^ on the same timescale can be assigned to the initial charge-separated excited vibrational state for the C≡C band. The second time component can be attributed to the additional energy transfer process that also occurs in the system, though with much lower efficiency. It is worth mentioning that this time constant (500 ps) is within the same order of magnitude as the energy transfer lifetime values found in TR-PL for the SDZ–TADF system (224 ps), supporting this assignment. After the first 500 ps, this charge-separated state disappeared, and only the main vibrational mode of the C≡C band at 2053 cm^–1^ remained. This deactivation or recombination is expected to occur on a timescale >4 ns (excited-state lifetime of the system). Interestingly, the remaining peak at 2053 cm^–1^ did not show any progressive shifts in time, as observed for pure Ac-SDZ. This result could be further evidence of the formation of the excited-state aggregate in pure Ac-SDZ and not in a mixture of Ac-SDZ and the TADF molecule. The charge separation and further electron donation to the acceptor decreased the vibrational C≡C mode’s lifetime in the Ac-SDZ–TADF mixture.

These results confirm the different behaviors of the Ac-SDZ molecule in the presence and absence of the TADF molecule in the excited state. The changes in the C≡C bond position and lifetime decay provide direct structural evidence of aggregate formation in the excited state for pure Ac-SDZ in a DMF solution and, most importantly, confirm ultrafast charge transfer from Ac-SDZ to the TADF molecule in the solution mixture.

### Density Functional Theory Calculations

**Figure 4:**
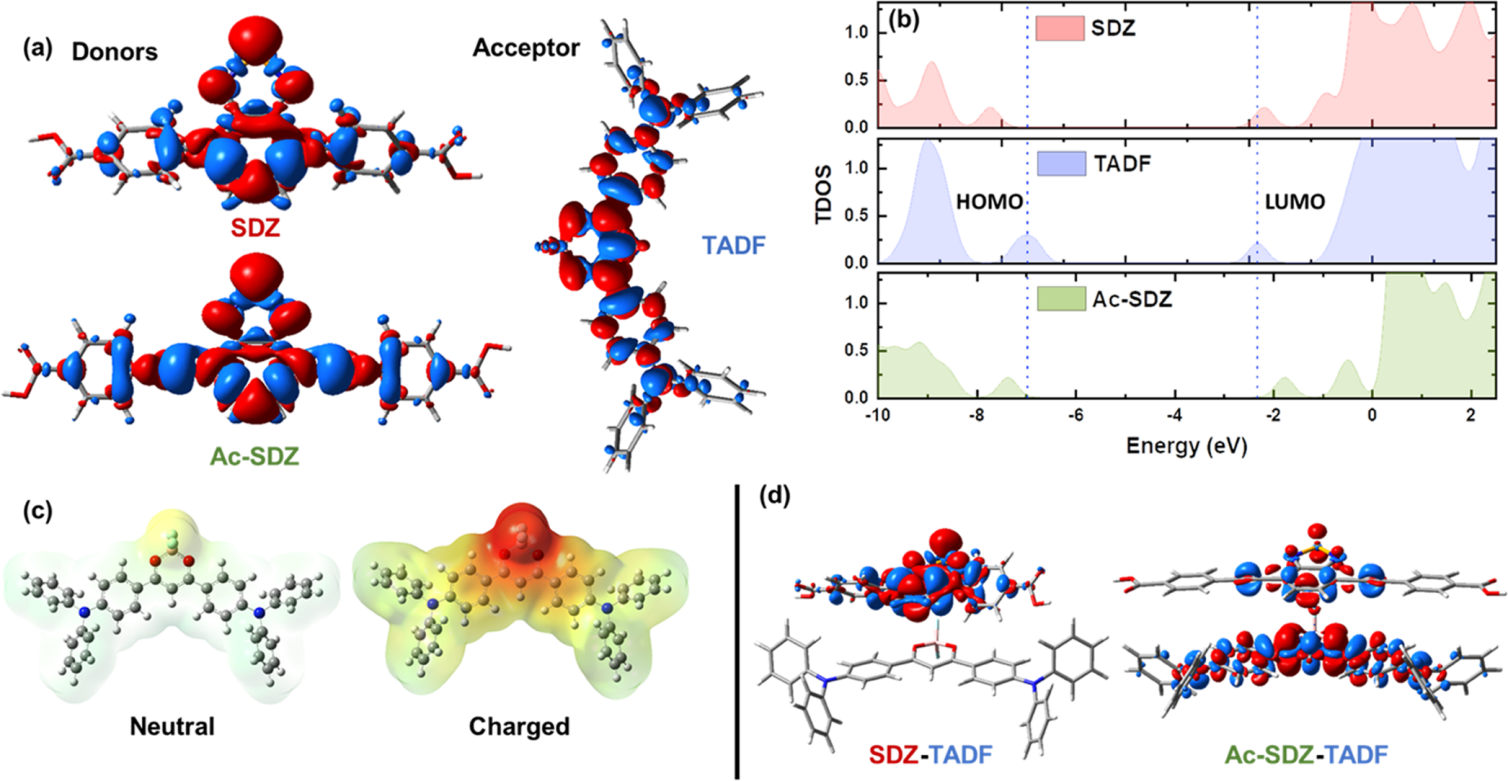
Electron density difference plot for the S_0_ → S_1_ transition in SDZ, Ac-SDZ, and TADF (a). Calculated total density of states (TDOS) for SDZ, TADF, and Ac-SDZ molecules. HOMO: highest occupied molecular orbital, LUMO: lowest unoccupied molecular orbital (b). Electrostatic potential surface for the neutral and negatively charged states of the TADF molecule (−0.1 to 0.1 au) (c). Electron density difference plot for the S_0_ → S_1_ transition in SDZ–TADF and Ac-SDZ–TADF (d). Red: +0.008 au, Blue: −0.008 au. Level of theory: CAM-B3LYP/6-311G++(d,p)/IEF-PCM: dichloromethane.

Based on all of the aforementioned experimental results, we performed DFT and TD-DFT calculations to gain further insight into the mechanistic details of the excited-state reaction for both donor–acceptor systems. First, by analyzing the molecules individually (Figure 4a), we observed that for both Ac-SDZ and SDZ, the photoinduced electronic transitions were localized in the central benzoselenadiazole core. It is worth mentioning that in the Ac-SDZ molecule, C≡C shows evidence of electron donor characteristics upon light excitation. Moreover, the TADF molecule also shows a delocalized electronic transition in its aromatic rings. Being in this regime, a further illustration of the energy levels can be observed in the total density of states (TDOS) plot in Figure 4b. By comparing the position of the donor energy levels with that of the acceptor, we can see that the SDZ and TADF molecules possess LUMO levels with similar energies (Δ*E* = 4 meV), which is a desirable feature during energy transfer processes. On the other hand, the LUMO for Ac-SDZ is at 0.54 eV, higher than that of the TADF molecule, which supports the feasibility of electron transfer from Ac-SDZ to the TADF unit. This can also be clearly observed from the HOMO–LUMO energy-level diagram in Figure S10.

Moreover, the electrostatic potential surfaces in Figure 4c clearly indicate the distribution of charge density in the TADF molecule before and after electron transfer. We can observe the accumulation of charge near the boron region. This evidence can be complemented with the optimized geometries for the donor–acceptor dyads, where this electron-receiving portion interacts with the benzoselenadiazole ring in both SDZ and Ac-SDZ molecules through π–π interactions, with distances of 4.75 and 4.86 Å, respectively (Figures 4d, S11, and S12). Finally, the electron density difference plot shown in Figure 4d for the D–A pairs clearly indicates that in the SDZ–TADF system, the transition to the excited state is localized exclusively to the SDZ molecule, meaning that there is no interaction between the two moieties after the electronic transition takes place. In contrast, the Ac-SDZ–TADF system undergoes a transition that involves electron density reorganization in both fragments, with notable electron-loss regions around the C≡C bonds, confirming that electron transfer takes place from the Ac-SDZ molecule to the TADF molecule, as extracted from the fs-Mid-IR TA kinetic traces in Figure 3c. These findings corroborate the crucial role of the C≡C bond in tuning the energy/charge transfer processes, as confirmed by our steady-state and time-resolved spectroscopic results.

## Conclusions

In this work, we present a simple way to control energy and electron transfer processes at the interface of bifluorescence systems by discrete changes in chemical structure. The two donors employed here were designed to possess a discrete structural difference via the addition of an acetylene bridge. Our results indicate that the SDZ–TADF system exhibited efficient energy transfer on the scale of hundreds of picoseconds. In contrast, when an acetylene bridge was added to the donor molecule, the system formed by Ac-SDZ–TADF showed charge transfer, as evidenced by steady-state and time-resolved experiments. Mid-IR transient absorption measurements highlighted the importance of the C≡C bond in the charge transfer process and indicated that this process takes place on the picosecond timescale. Additionally, we investigated both donor–acceptor dyads by DFT and TD-DFT, examining energy-level comparisons and electron density differences to confirm that energy transfer from both donors to the TADF molecule was possible; in the case of Ac-SDZ, an efficient charge transfer process could also occur. The calculations clearly indicate that this charge transfer occurs from the C≡C bond to the central ring of the TADF molecule. Our findings in this study show that a slight modification of the chemical structure by including electron-donor/-withdrawing groups induces significant implications for fine-tuning photoinduced processes in donor–acceptor systems involving TADF molecules to improve their optical properties for OLEDs, X-ray imaging scintillators, and fluorescence sensing. Finally, we are currently exploring these linkers as MOF building blocks and evaluating their optical properties and potential applications in X-ray imaging and visible light communications.

## Supporting Information Available

Detailed synthetic procedures and molecule characterizations, additional steady-state and time-resolved spectroscopic data, TCSPC fitting parameters, calculated IR spectra, and DFT-optimized geometries (PDF)
