# Supplementary material for: Tunable Photoinduced Charge Transfer at the Interface between Benzoselenadiazole-Based MOF Linkers and Thermally Activated Delayed Fluorescence Chromophore
Source: J Phys Chem B. 2023 Feb 21;127(8):1819–27. doi: 10.1021/acs.jpcb.2c08844 (PMC9986871; doi:10.1021/acs.jpcb.2c08844)
Supplement: Supplementary file 1 — jp2c08844_si_001.pdf [file jp2c08844_si_001.pdf]

Supporting information for

Tunable Photoinduced Charge Transfer at the Interface between Benzoselenadiazole-Based MOF Linkers and Thermally Activated Delayed Fluorescence Chromophore

Shorooq A. Alomar,[1] Luis Gutiérrez-Arzaluz,[1] Issatay Nadinov,[1,3] Ru He,[4] Xiaodan Wang,[4] Jian-Xin Wang,[1] Jiangtao Jia,[2] Osama Shekhah,[2] Mohamed Eddaoudi,[2] Husam N. Alshareef ,[3] Kirk S. Schanze,[4] and Omar F. Mohammed[*,1]*

[1]Advanced Membranes and Porous Materials Center and KAUST Catalysis Center, Division of Physical Science and Engineering, King Abdullah University of Science and Technology, Thuwal 23955-6900, Kingdom of Saudi Arabia

[2]Functional Materials Design, Discovery and Development Research Group (FMD), Advanced Membranes and Porous Materials Center (AMPMC), Division of Physical Sciences and Engineering (PSE), King Abdullah University of Science and Technology (KAUST), Thuwal 23955-6900, Saudi Arabia

[3]Materials Science and Engineering, Division of Physical Sciences and Engineering (PSE), King Abdullah University of Science and Technology (KAUST), Thuwal 23955-6900, Saudi Arabia

[4]Department of Chemistry, University of Texas at San Antonio, San Antonio, Texas 78249, United States.

Contents

Detailed Synthetic Procedures and Characterization....................S2

Steady-State Spectroscopy....................S7

Time-resolved Spectroscopy....................S8

DFT Calculations....................S9

Detailed Synthetic Procedures and Characterization

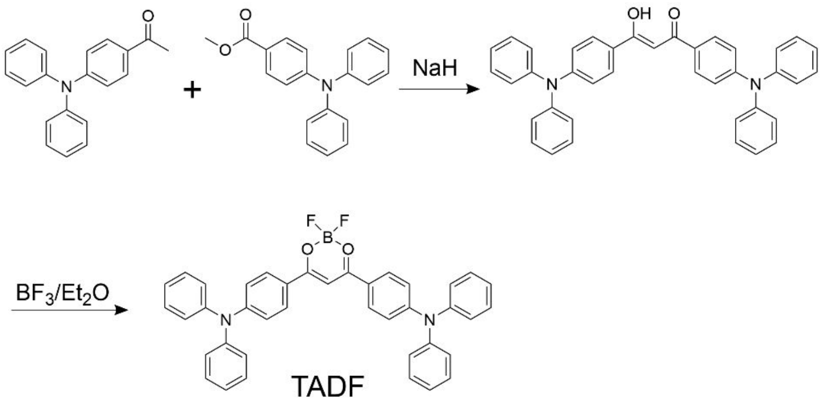

Scheme S1. Synthesis procedures of TADF.

Synthesis of **TADF** molecule:[1]

1-(4-(diphenylamino)phenyl)ethanone (1.0 g, 3.5 mmol) was dissolved in 10 mL anhydrous THF in a high-pressure tube and bubbled with N_2 for 5 min. Later, NaH (57-63% oil dispersion, 800 mg, 20.0 mmol) and methyl 4-(diphenylamino)benzoate (1.1 g, 3.5 mmol) were added. The reaction mixture was stirred at 60 °C for 24 h under N_2 atmosphere. The reaction mixture was then cooled to room temperature and carefully quenched with 100 mL of deionized water. The pH was adjusted to 3 with diluted HCl (aq), and the product was extracted with CH_2Cl_2. The combined organic phase was dried over anhydrous Na_2SO_4, filtrated, and the solvent was evaporated. The result was used directly for the next step without further purification. To a solution of the product of the previous step (30 mL CH_2Cl_2), Et_3N (2.0 mL) and BF_3/Et_2O (2.0 mL) were added. The resulting reaction mixture was stirred for 2 h at room temperature in the dark, followed by adding 100 mL of water. The organic layer was collected, washed with saturated aqueous NH_4Cl, and dried over anhydrous Na_2SO_4, followed by filtration and evaporation of the solvent. Column chromatography using CH_2Cl_2/petroleum ether (1:1, v/v) as the eluent to afford 1.2 g of **A (TADF)** as red solid (yield: 66%). ^{1}H NMR (400 MHz, $CDCl_3$): δ 7.92 (d, J = 8.8 Hz, 4 H), 7.36 (t, J = 7.7 Hz, 8 H), 7.20 (t, J = 8.0 Hz, 12 H), 6.98 (d, J = 8.9 Hz, 4 H), 6.87 (s, 1 H). ^{13}C NMR (100 MHz, $CDCl_3$): δ 178.9, 153.7, 145.8, 130.5, 129.9, 126.6, 125.6, 123.5, 118.9, 91.1. HRMS: calc. for [$M+H^+$] 607.2363, found: 607.2366.

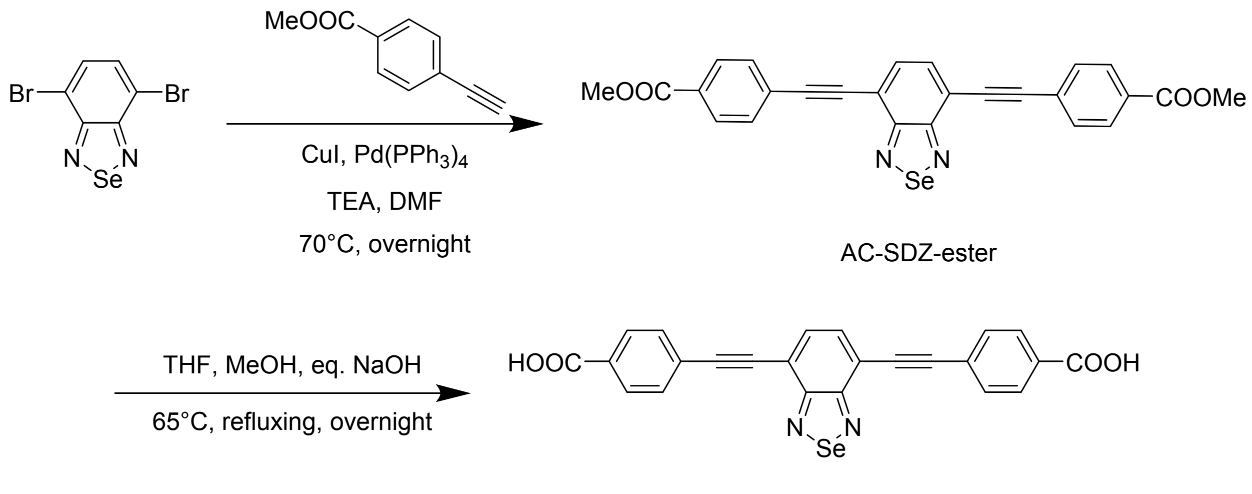

AC-SDZ

Scheme S2. Synthesis procedures of Ac-SDZ.

Synthesis of **Ac-SDZ-ester**:

Methyl 4-ethynylbenzoate (352 mg, 2.20 mmol, 2.5 eq) was dissolved in DMF (anhydrous, 170 mL) and degassed for 15 min. 4,7-Dibromo-2,1,3-benzoselenadiazole (300 mg, 0.88 mmol, 1 eq, synthesized based on reported paper[3]), TEA (30 mL), CuI (20 mg, 0.11 mmol) and $Pd(PPh_3)_4$ (50 mg, 0.07 mmol) were added to the solution and degassed for another 10 min. The mixture was stirred at 70 °C overnight under N_2 atmosphere. After cooling to room temperature, DI water (80 mL) was added to get the precipitants of the crude product. Separated the precipitants with Büchner funnel under vacuum, washed with a large amount of DI water and hexane until the filtrate became colorless. Washed with several drops of DCM to remove unreacted starting materials. The product left on filter paper was dried at 30 °C under vacuum to give a brown solid (430.00 mg, 0.86 mmol, 98 %). ^{1}H NMR (500 MHz, $CDCl_3$): δ/ppm 3.89 (s, 6H), 7.79 (d, 4H, J = 10 Hz), 7.81 (s, 2H), 8.01 (d, 4H, J = 10 Hz).

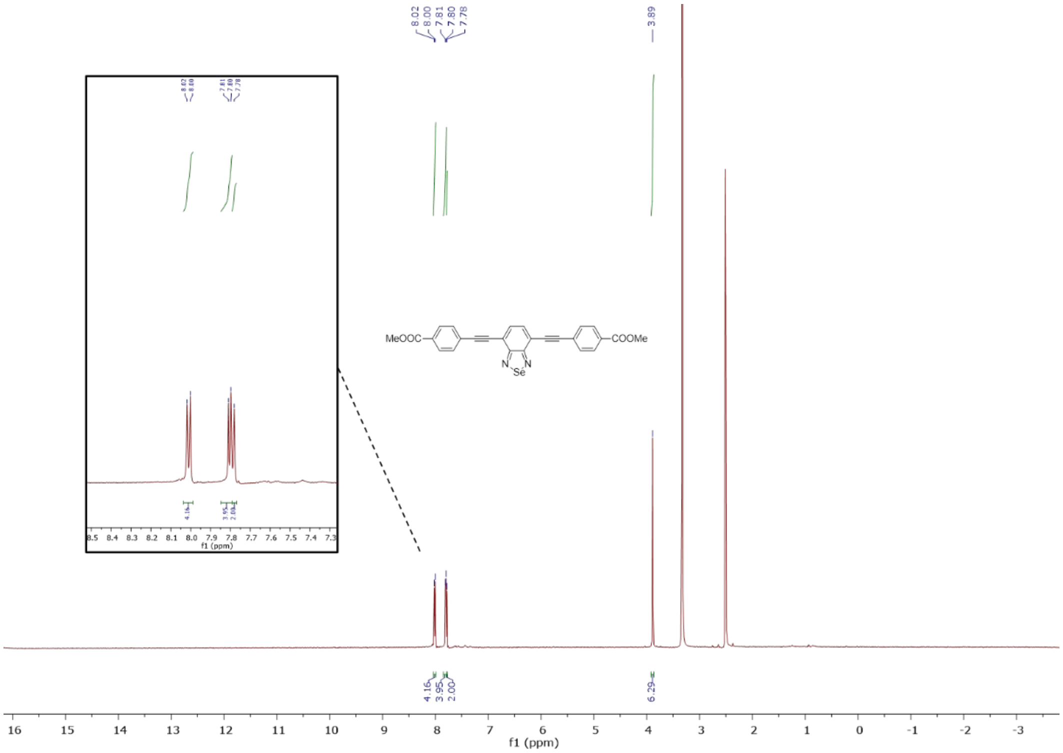

Figure S1. ^{1}H NMR of **Ac-SDZ-ester** (500 MHz, $CDCl_3$).

Synthesis of **Ac-SDZ**:

Suspended **AC-SDZ-ester** (200 mg, 0.40 mmol, 1 eq) in THF (anhydrous, 60 mL). Sodium hydroxide (2 g, 50 mmol, 113 eq) was dissolved in DI water (8 mL) to get an alkaline solution. Added MeOH (10 mL) and the alkaline solution into the ester suspension, heated the whole reaction system at 65 °C overnight under reflux. After cooling to room temperature, added DI water (50 mL) and 2 M HCl (eq) until pH~2 and the product precipitated. Separated the product by centrifugation and rinsed several times with DI water until neutral pH. An orange solid (149.5 mg, 0.32 mmol, 79 %) was obtained after drying at 30 °C under vacuum. ^{1}H NMR (500 MHz,

DMSO-d_6): δ/ppm 7.75 (d, 4H, J = 10 Hz), 7.90 (s, 2H), 8.03 (d, 4H, J = 5 Hz), 13.21 (br s, 2H). ^{13}C NMR (125 MHz, DMSO-d_6): δ/ppm 89.36, 96.15, 118.47, 126.50, 130.18, 131.75, 132.13, 133.25. HRMS (ESI): calculated for $C_{24}H_{14}N_2O_4Se^{2+}$ [M^{2+}]: 473.0036, experimental 473.0027.

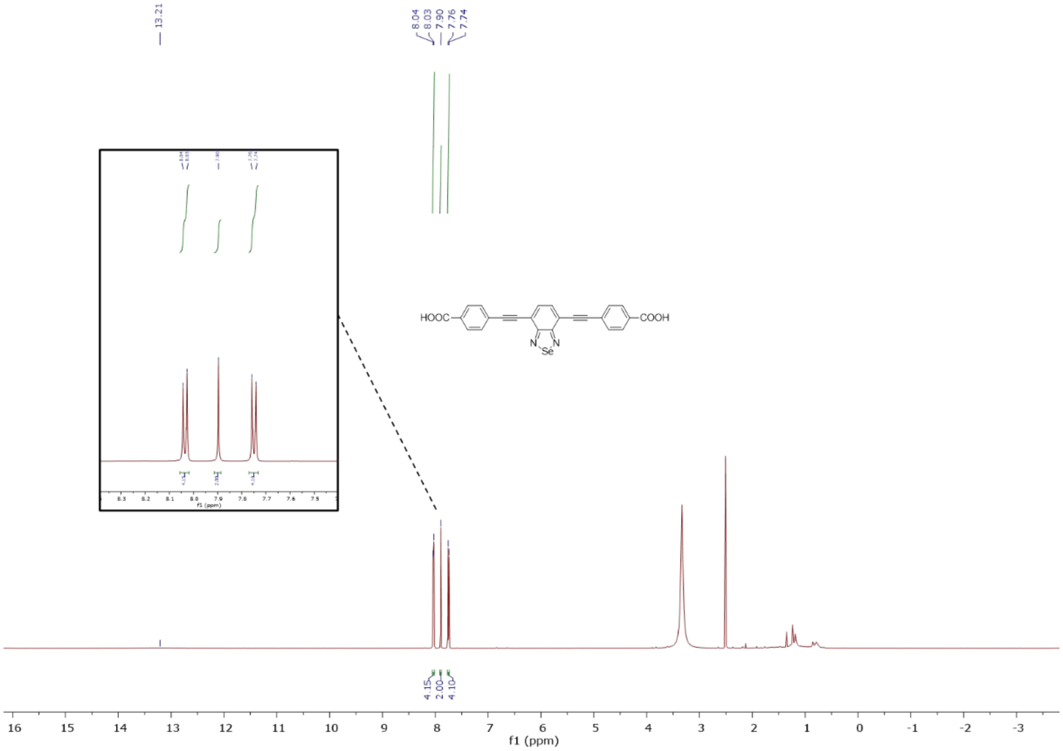

Figure S2. ^{1}H NMR of **Ac-SDZ** (500 MHz, DMSO-d_6).

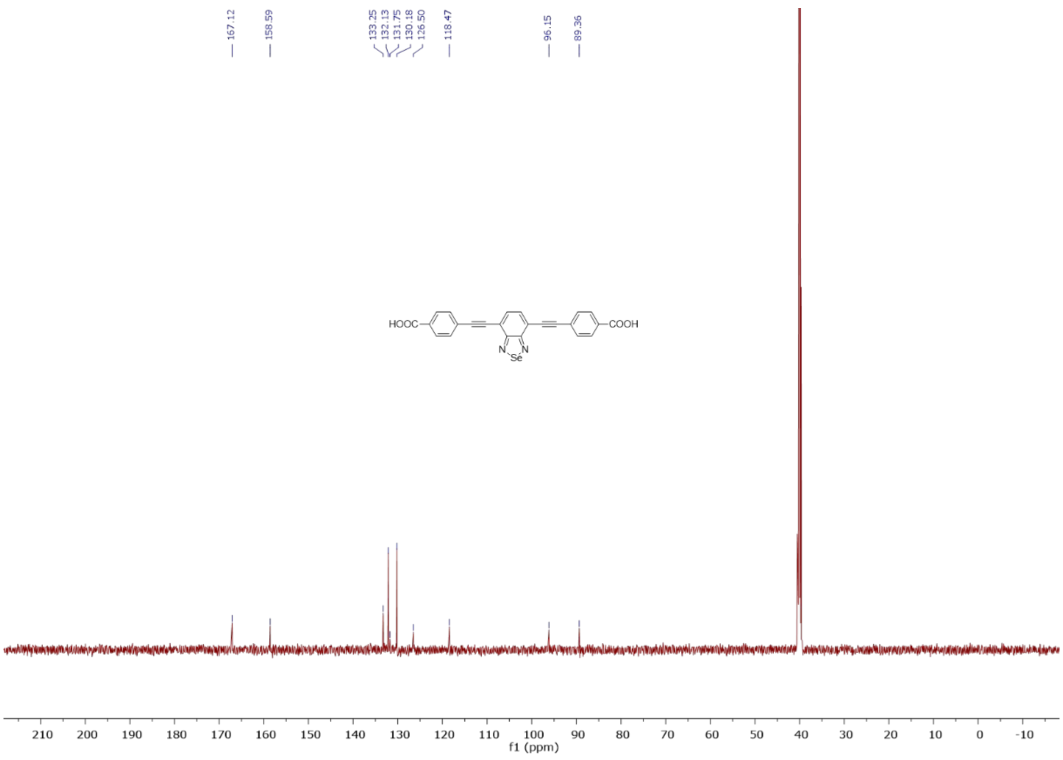

Figure S3. ^{13}C NMR of **Ac-SDZ** (125 MHz, DMSO-d_6).

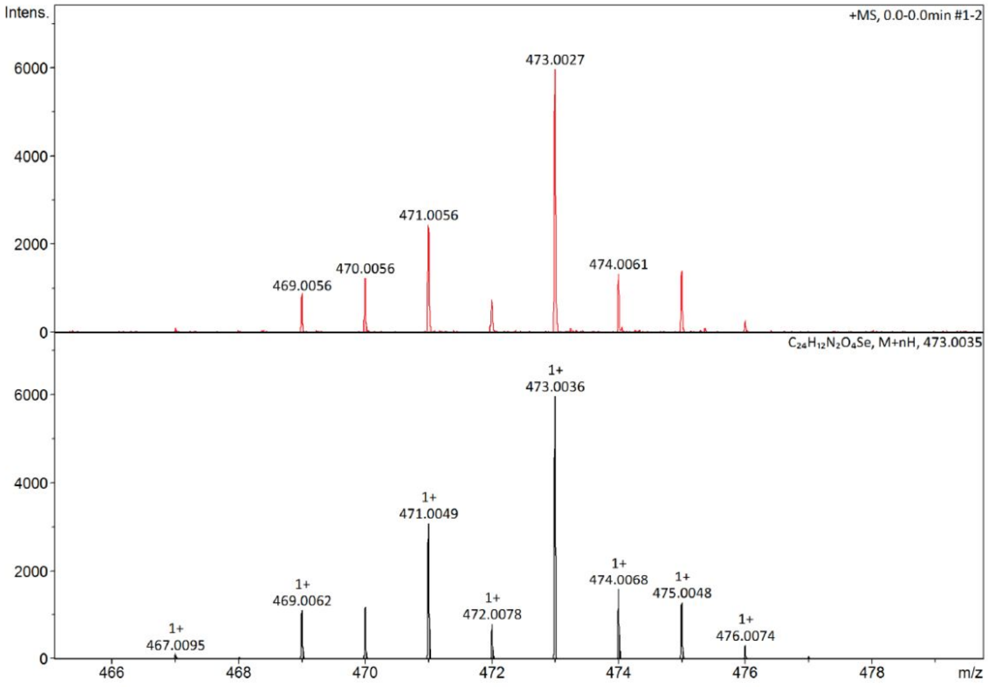

Figure S4. ESI-MS of **Ac-SDZ**.

Steady-State Spectroscopy

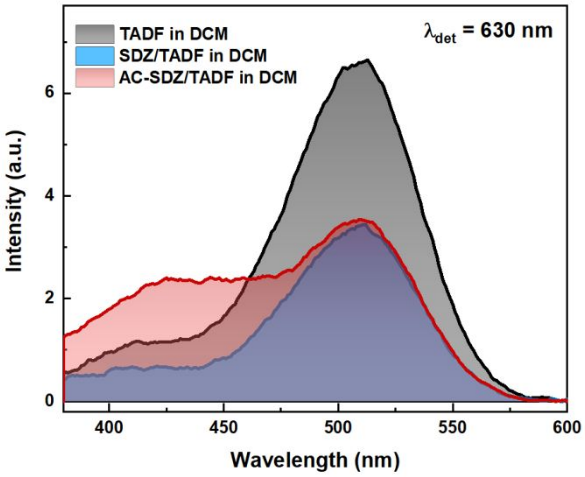

Figure S5. PL excitation spectra for the TADF molecule and in the presence of the SDZ and Ac-SDZ donors in DCM solution.

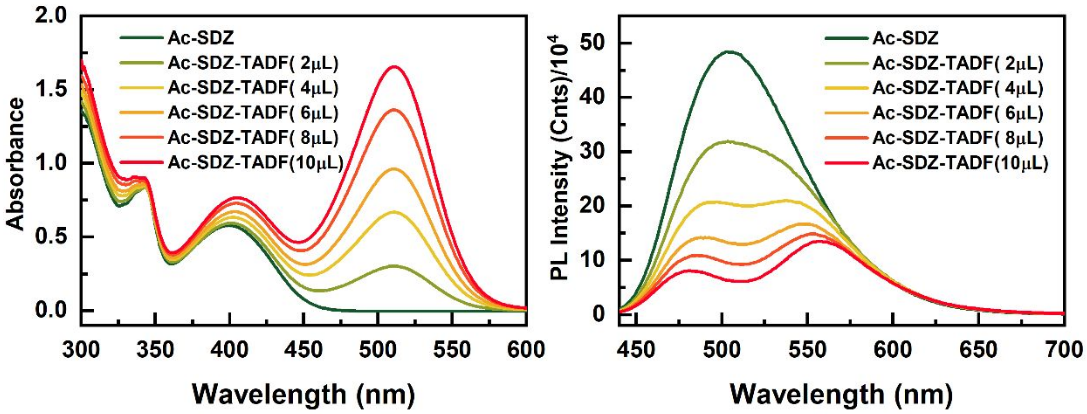

Figure S6. Absorption and emission spectra for molecular systems of Ac-SDZ in DMF and after the increases in the TADF concentration.

Time-resolved Spectroscopy

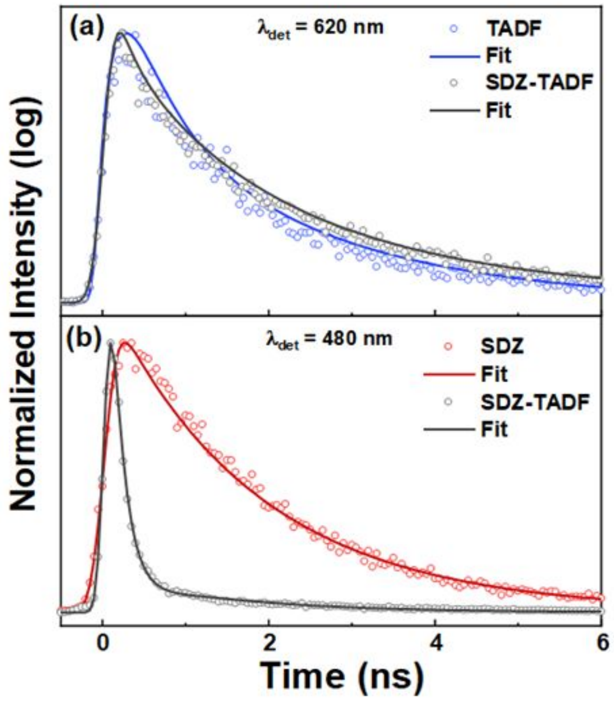

Figure S7. Fluorescence decays for pure TADF and SDZ and the mixture detected (a) at 620 nm and (b) at 480 nm. λ_{exc} = 350 nm

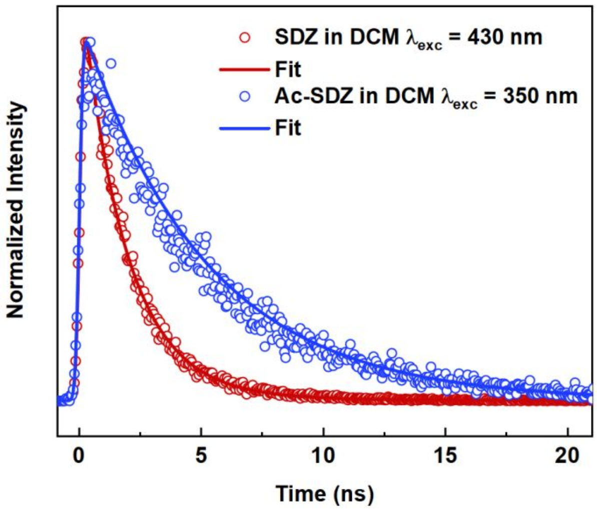

Figure S8. PL decays for donors SDZ and Ac-SDZ in DCM solutions monitored at their emission maxima.

Table S1. Time-resolved PL fitting parameters for TADF acceptor, SDZ and Ac-SDZ donors; and the SDZ/TADF and Ac-SDZ/TADF systems.

System	Parameters
**TADF in DCM** λ_{exc} **= 350 nm** λ_{emi} **= 620 nm**	0.2 ns (46.6 %); 0.5 ns (30.2 %); 2.2 ns (23.2 %)
**SDZ/TADF in DCM** λ_{exc} **= 350 nm** λ_{emi} **= 620 nm**	0.2 ns (27.9 %); 1.6 ns (50.5 %); 4.0ns (21.5 %)
**SDZ/TADF in DCM** λ_{exc} **= 350 nm** λ_{emi} **= 480 nm**	0.2 ns (99.8 %); 1.4 ns (0.2 %)
**SDZ in DCM** λ_{exc} **= 430 nm** λ_{emi} **= 485 nm**	1.86 ns
**Ac-SDZ/TADF in DCM** λ_{exc} **= 350 nm** λ_{emi} **= 550 nm**	1.9 ns (32.6%); 2.2ns (44.1 %); 5.9 ns (23.2 %)
**TADF in Toluene** λ_{exc} **= 350 nm** λ_{emi} **= 550 nm**	2.3 ns (75.5 %); 3.5 ns (24.3 %)

DFT Calculations

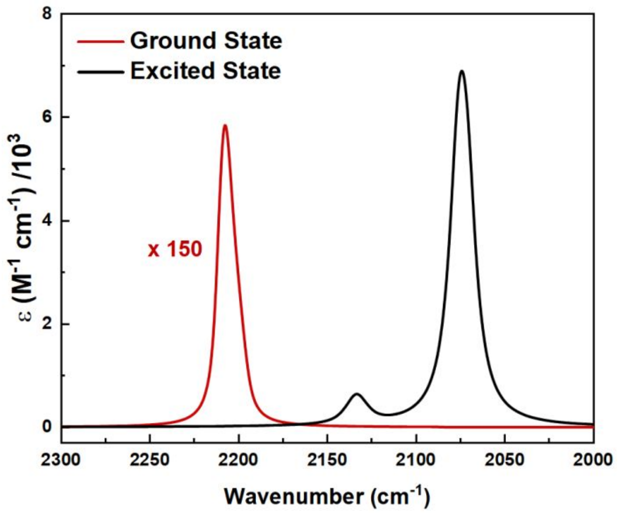

Figure S9. Calculated IR absorption spectra for the Ac-SDZ molecule in the ground and excited states. Level of theory: CAM-B3LYP/6-311G++ (d,p)/IEF-PCM: dichloromethane.

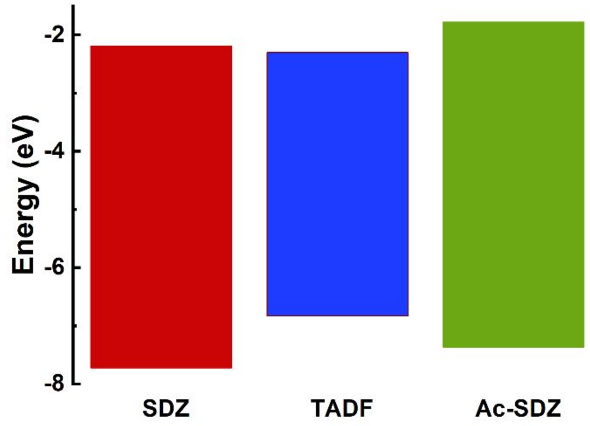

Figure S10. Calculated HOMO-LUMO energy gap diagram for the SDZ, TADF, and Ac-SDZ molecules. Level of theory: CAM-B3LYP/6-311G++ (d,p)/IEF-PCM: dichloromethane.

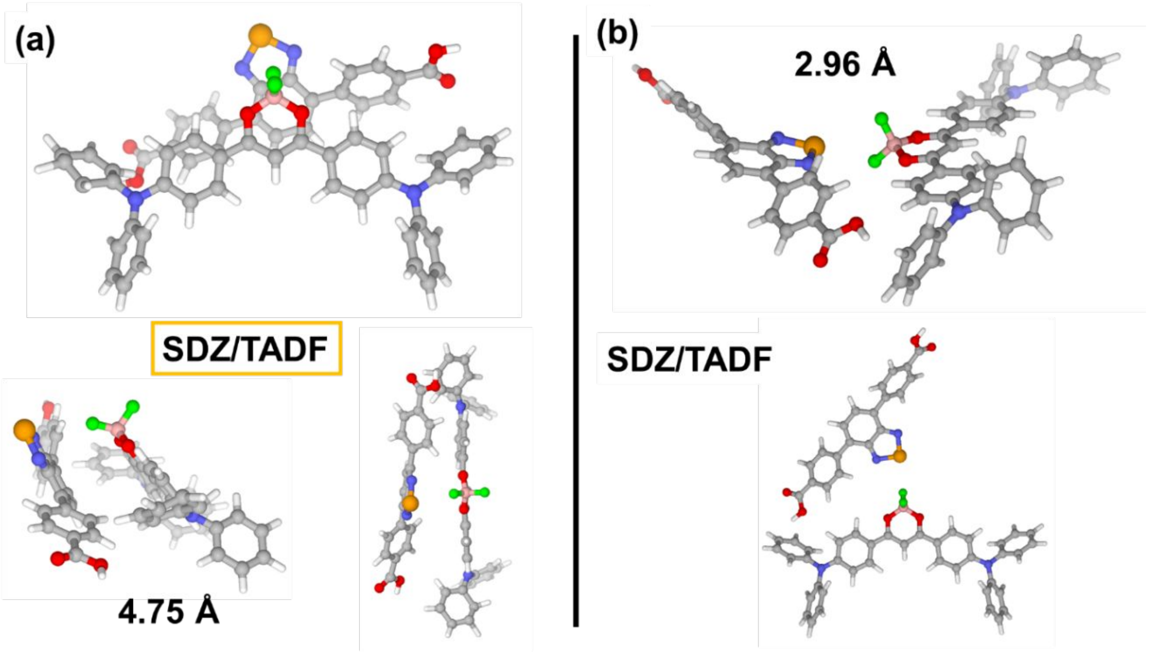

Figure S11. Ground state optimized SDZ/TADF pairs in (a) stacked and (b) front-to-front configurations. The most stable configuration is highlighted. Level of theory: CAM-B3LYP/6-311G++ (d,p)/IEF-PCM: dichloromethane.

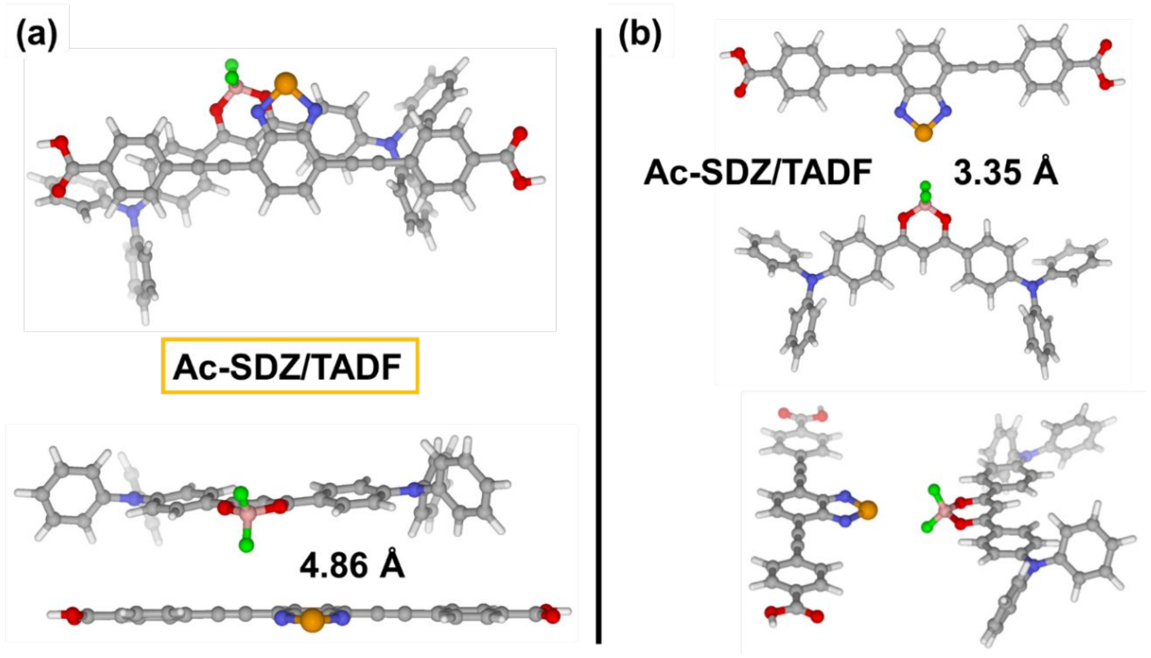

Figure S12. Ground state optimized Ac-SDZ/TADF pairs in (a) stacked and (b) front-to-front configurations. The most stable configuration is highlighted. Level of theory: CAM-B3LYP/6-311G++(d,p)/IEF-PCM: dichloromethane.

References

1. Wang, J.-X.; Zhang, H.; Niu, L.-Y.; Zhu, X.; Kang, Y.-F.; Boulatov, R.; Yang, Q.-Z., Organic Composite Crystal with Persistent Room-Temperature Luminescence Above 650 nm by Combining Triplet-Triplet Energy Transfer with Thermally Activated Delayed Fluorescence. *CCS. Chem.* **2020**, *2*, 1391-1398.
